# Direct observation and identification of nanoplastics in ocean water

**DOI:** 10.1126/sciadv.adh1675

**Published:** 2024-01-26

**Authors:** Seunghyun Moon, Leisha M. A. Martin, Seongmin Kim, Qiushi Zhang, Renzheng Zhang, Wei Xu, Tengfei Luo

**Affiliations:** ^1^ Department of Aerospace and Mechanical Engineering, University of Notre Dame, Notre Dame, IN 46556, USA.; ^2^ Department of Life Sciences, Texas A&M University, Corpus Christi, TX 78412, USA.; ^3^ MNT SmartSolutions, 204 Bryn Mawr, Albuquerque, NM 87106, USA.; ^4^ Department of Chemical and Biomolecular Engineering, University of Notre Dame, Notre Dame, IN 46556, USA.; ^5^ Center for Sustainable Energy of Notre Dame (ND Energy), University of Notre Dame, Notre Dame, IN 46556, USA.

## Abstract

Millions of tons of plastics enter the oceans yearly, and they can be fragmented by ultraviolet and mechanical means into nanoplastics. Here, we report the direct observation of nanoplastics in global ocean water leveraging a unique shrinking surface bubble deposition (SSBD) technique. SSBD involves optically heating plasmonic nanoparticles to form a surface bubble and leveraging the Marangoni flow to concentrate suspended nanoplastics onto the surface, allowing direct visualization using electron microscopy. With the plasmonic nanoparticles co-deposited in SSBD, the surface-enhanced Raman spectroscopy effect is enabled for direct chemical identification of trace amounts of nanoplastics. In the water samples from two oceans, we observed nanoplastics made of nylon, polystyrene, and polyethylene terephthalate—all common in daily consumables. The plastic particles have diverse morphologies, such as nanofibers, nanoflakes, and ball-stick nanostructures. These nanoplastics may profoundly affect marine organisms, and our results can provide critical information for appropriately designing their toxicity studies.

## Teaser

Direct visualization of nanoplastics reveals their diverse morphologies and widespread presence in our environmental water.

## INTRODUCTION

Annual global production of plastics exceeds 400 million tons (*1*). Disposing of these products is a major challenge for the ecosystem as 79% of them are accumulated in landfills or the natural environment, and 0.4 million to 4 million tons enter the oceans (*1**–**4*). Although consensus has not been reached on the exact size ranges of “microplastics” and “nanoplastics,” some have defined microplastics as ranging from 1 to 5000 μm and nanoplastics with a size less than 1 μm. Microplastics are abundant in the environment and have been observed in the open ocean (*5**–**7*), the Great Lakes (*8*), and rivers (*5**–**7*, *9*). Over time, plastic products degrade into even smaller particles (*10*, *11*), and this has been observed under laboratory conditions (*12**–**14*). For instance, disposable polystyrene (PS) coffee cup lids only take about 2 months to decompose into nanoplastics in a weathering chamber (*15*).

Existing techniques used for nanoplastics detection are mainly based on pyrolysis-coupled gas chromatography–mass spectrometry (Pyr-GC/MS), where extensive concentration steps using ultrafiltration are needed to measure the trace amount of nanoplastics in the ocean water (*16*, *17*). While quantitative, they cannot provide information about nanoplastics, such as sizes and morphologies. Raman spectroscopy combined with scanning electron microscopy (SEM) has been used to show evidence of the release of nanoplastics from recycled polyvinyl chloride (PVC) powders, but the observation was not from environmental water (*18*). A Raman tweezer technique was used to capture and identify engineered plastic particles spiked in seawater, but the follow-on optical microscopic observation was not able to provide sufficient resolution for the morphology of nanoplastics (*19*). As previously suggested by other studies, the toxicity of micro- and nanoplastics to living organisms is found to be inversely related to particle size and morphology (*11*, *20*). For example, when laboratory-synthesized PS particles are ingested, nanoparticles (NPs) have a more negative impact on growth and reproduction than microparticles (*21*). In addition, in a laboratory setting, while microplastics were not found in fish brains after exposure (*22*), nanoscale particles were observed to cross the blood-brain barrier and accumulate in fish brains, causing behavioral disorders (*23*) and oxidative DNA damage (*24*). Therefore, the direct visualization of nanoplastics in the environment is highly desired.

**Fig. 1.**
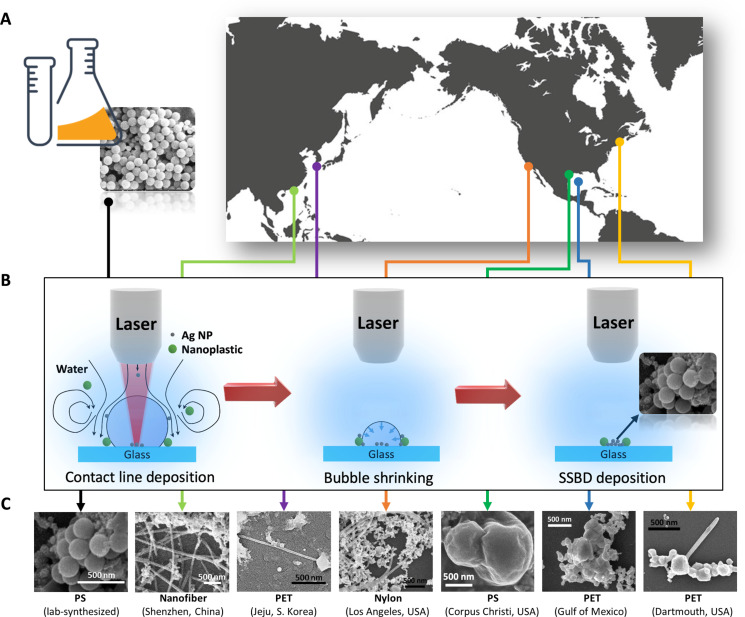
Direct observation of nanoplastics from ocean water around the world. (**A**) Locations of seawater collection on the coastlines of China, South Korea, the United States, and in the Gulf of Mexico (location coordinates in table S1), together with a laboratory reference PS colloidal water sample. (**B**) Schematics of the SSBD process, where a thermal bubble generated by laser heating creates a thermofluidic flow to collect suspended particles and deposit them to a high-density spot (right inset) after the bubble shrinks and vanishes. (**C**) SEM images of nanoplastics observed from different water samples, which show the diverse morphologies of nanoplastics in environmental water and how they are distinct from laboratory-synthesized plastic NPs (leftmost picture).

Here, we leverage a unique shrinking surface bubble deposition (SSBD) technique. The technique leverages the plasmonic heating of silver (Ag) NPs, which are mixed with environmental water samples, upon laser excitation to generate a bubble and use the resulting thermofluidic flow to greatly concentrate the suspended particles in water and deposit them on a substrate for observation and characterization. We use SEM to clearly visualize the morphology of the nanoplastic particles, use energy-dispersive x-ray (EDX) spectroscopy to confirm their carbon nature, and use surface-enhanced Raman spectroscopy (SERS) to identify their polymer chemistry. This method enables the direct visualization and identification of nanoplastics in water samples collected from seven different locations across two oceans (Fig. 1A and table S1).

## RESULTS

### SSBD technique

In the SSBD process, the as-collected seawater samples are mixed with silver (Ag) NP suspensions (0.02 mg/ml, Thermo Fisher Scientific) with a 1:4 volume ratio. A laser is then directed into the aqueous sample, and due to the laser heating of the plasmonic Ag NPs, a thermal bubble is generated on the surface of a glass substrate (Fig. 1B and fig. S1) (*25**–**28*). The thermofluidic flow around the bubble draws suspended particles, regardless of their types (e.g., nanoplastics and Ag NPs), to the bubble surface and deposits them to the three-phase contact line (Fig. 1B and fig. S2) (*26*, *27*, *29**–**35*). When the surface bubble grows to ~40 μm in diameter, we stop laser heating to allow the bubble to shrink until it vanishes. As the bubble shrinks, the contact line contracts, piling the collected particles there into a high-density spot that sticks to the surface due to van der Waals interactions, which greatly increases the possibility of finding them in SEM (Fig. 1B, inset). The detailed mechanism of particle deposition in plasmonic solutions, including the thermal capillary flow around the bubble and the shrinking-induced contact line contraction, has been previously elucidated in the literature including our work (*26*, *30*, *31*, *34*). During the SSBD process, the plasmonic Ag NPs in the suspension are co-deposited with nanoplastics, enabling the SERS effect that enhances the Raman signal by orders of magnitude to allow the identification of the chemistry of trace amounts of nanoplastics (see Materials and Methods) (*36*, *37*). Without the co-deposited Ag NPs, no clear Raman signals of nanoplastics could be detected (fig. S3). Therefore, the plasmonic Ag NPs play a dual role, contributing to both the formation of the plasmonic surface bubble, which collects, concentrates, and deposits the nanoplastics, and the surface-enhanced Raman SERS effect. We note that care has been taken during the water collection and sample preparation process by using glassware to eliminate possible contamination from unwanted plastics (see Materials and Methods). To ensure that the Ag NPs solution does not contribute to the plastic signals, we have also performed SSBD for Ag NPs mixed with plain NaCl solution (3 wt %) and found no Raman peaks corresponding to plastics (see fig. S4A).

### Observation of nylon nanofibers

**Fig. 2.**
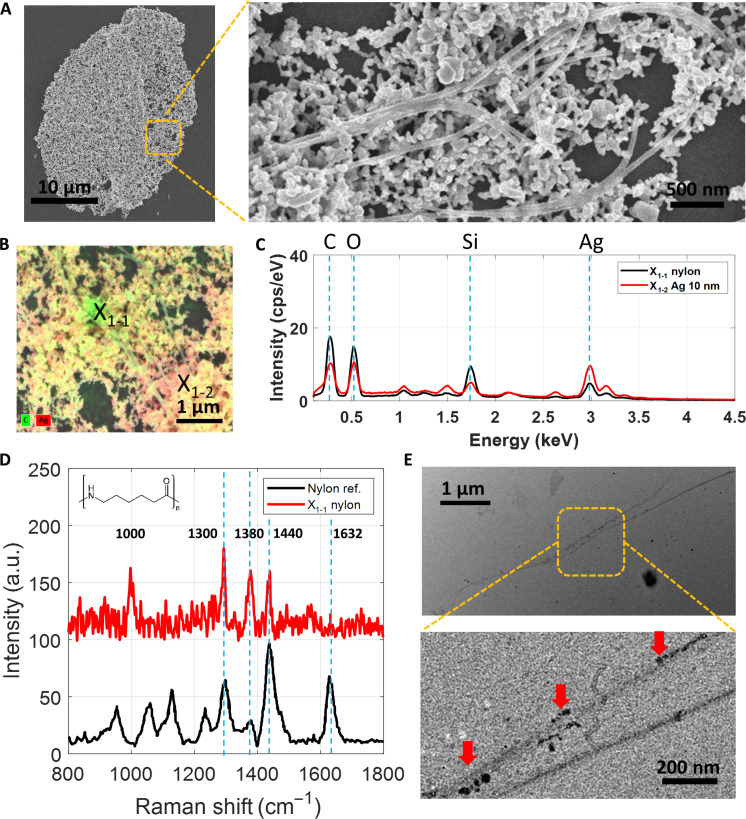
Observation of nylon nanofiber in water from Long Beach, CA. (**A**) SEM images of the whole SSBD spot (left) and nanofibers co-deposited with Ag NPs (right). (**B**) EDX elemental mapping overlaid with the SEM image, and (**C**) contrasting spectra show that the nanofibers are of a carbon nature. (**D**) The obtained SERS spectrum (red) shows several peaks corresponding to nylon reference peaks (black) (*19*). (**E**) Transmission electron microscopy (TEM) images of nanofibers show hetero-aggregation and adsorption of nanoplastics, as indicated by the red arrows. The TEM sample was prepared using SSBD in pristine seawater without Ag NPs, where the carbon TEM grid absorbed laser energy and generated a bubble.

In the seawater from Long Beach, CA, we found nanofibers in the SSBD spot (Fig. 2A) whose diameters are ~20 nm and lengths are longer than several micrometers. The aggregates around the nanofibers are Ag NPs co-deposited during the SSBD process. The corresponding EDX elemental mapping in Fig. 2B shows uniform carbon elements along the nanofibers surrounded by Ag NPs. Two selected points of EDX spectra are compared, with one from the nanofiber-rich (X_1–1_) region and the other from the Ag-rich (X_1–2_) region (Fig. 2C). The differences in the two spectra confirm that the nanofibers contain carbon (x-ray energy of 0.277 keV) and show they also contain oxygen (0.525 keV). We note that the silicon peak is from the glass substrate.

The obtained SERS spectrum (Fig. 2D) clearly shows peaks of weak amide I (C=O stretch) at 1632 cm^−1^, strong CH_2_ bending at 1440 cm^−1^, wagging at 1380 cm^−1^, and twisting at 1300 cm^−1^ (*38*). Many of these peaks correspond well to the signature of the nylon (polycaprolactam) reference (*19*). Not every nylon peak shows up in our SERS spectrum, which may be attributed to the fact that the peaks can be weakened or absent when the Raman polarizability component is tangential to metal surfaces, which are from the Ag NPs in our case (*39*). The unidentified peak at 1000 cm^−1^ may be attributed to the reported hetero-aggregation or adsorption of nanoplastics (*19*, *40*), which is confirmed by our transmission electron microscope (TEM) characterization (Fig. 2E). Considering the shape of the nanostructure from SEM, its constituent atoms from EDX, and chemical signatures from SERS, we believe that the observed nanofiber is nylon (*19*). These nylon nanofibers in marine environments can be from industrial wastewater, abandoned dumps, fishing gear, or laundry wastewater (*41*). Gillibert *et al.* (*19*) used the Raman tweezer technique and detected nylon signals, but their optical image did not have sufficient resolution to visualize the morphology of the nylon fiber. In the water sample from Shenzhen, China, we have also observed similar nanofibers. We have confirmed that they are of a carbon nature, but their chemistry is not conclusively (fig. S5).

### Observation of PS Nanoplastics

**Fig. 3.**
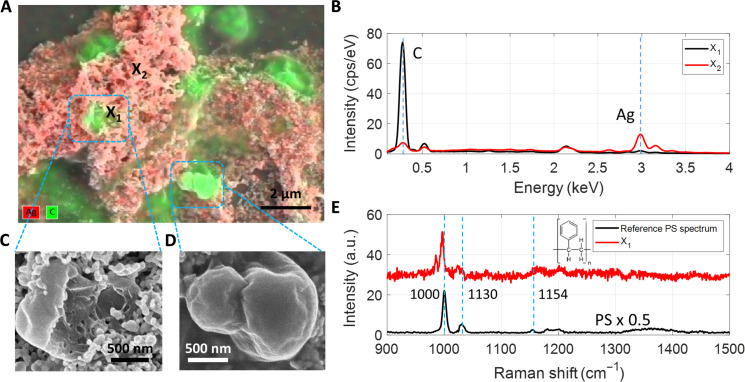
Observation of PS NPs from various locations. (**A**) EDX elemental map overlaid with the SEM image. Red and green indicate Ag and carbon elements, respectively. (**B**) Contrasting EDX spectra of two points respectively from carbon-rich and Ag reach regions. (**C**) SEM image of a flake-like plastic particle. (**D**) SEM image of a plastic particle with a neck. (**E**) SERS spectrum obtained from the X_1_ site in (A) compared against the reference Raman spectrum taken from a commercial coffee cup lid. Wider spectra are presented in fig. S6.

In the seawater collected from Corpus Christi, TX, we found several irregularly shaped particles. The EDX elemental mapping of the SSBD spot shows several carbon-rich regions (Fig. 3A), and Fig. 3B shows the contrast between the carbon-rich regions and the Ag-rich aggregates. SEM reveals that these are particles with certain dimensions less than 1 μm (Fig. 3, C and D). The irregular structure in Fig. 3C with submicrometer thickness appears to be a partially damaged flake which may result from wear or other types of degradation. The image of another structure (Fig. 3D) appears to be a particle undergoing division into two smaller ones or two particles that hetero-aggregated. The measured SERS spectrum has several peaks that match the reference peaks from PS (Fig. 3E and fig. S6). According to the report on nanoplastics in the North Atlantic subtropical gyre using Pyr-GC/MS (*42*), PS is an abundant polymeric species as suggested by the instrument, but our images reveal their real morphologies in the environmental water. It is noteworthy that the appearance of colloidal PS nanoplastics in the ocean differs markedly from those synthesized in the laboratory (Fig. 1C and fig. S7), which are routinely used for toxicity studies (*43*).

In many water samples, the PS signals were detected by Raman analysis, but no PS particles could be seen by SEM (figs. S6 and S4B and table S1). This may be caused by PS degradation into smaller styrene oligomers, as previously reported to be abundant in the ocean (*44*). These oligomers, however, are too small to be captured by SEM imaging techniques.

### Observation of polyethylene terephthalate nanostructures

**Fig. 4.**
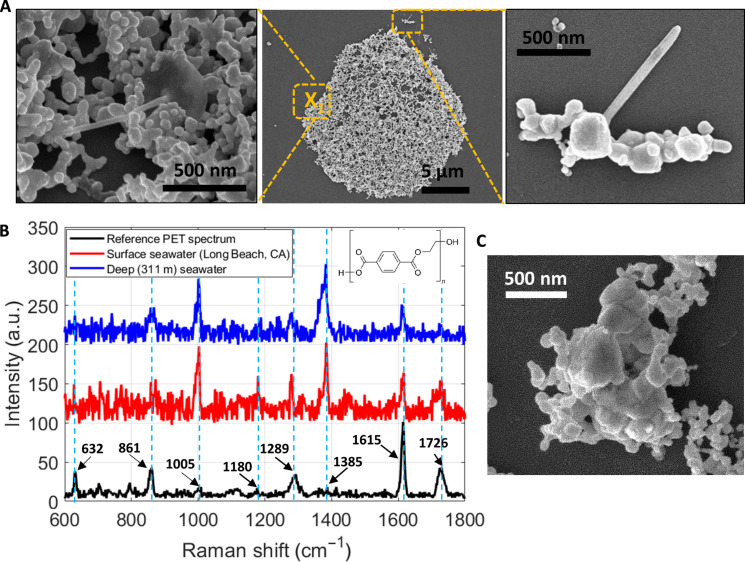
Observation of PET NPs in water from Long Beach, CA, and 311-m-deep seawater in an offshore location in the Gulf of Mexico. (**A**) SEM images of SSBD deposition (center) and ball-stick nanostructures for the Long Beach sample. X_1_ indicates the location characterized by SERS. (**B**) SERS spectra of the surface seawater sample from Long Beach (red) and 311-m-deep seawater from the Gulf of Mexico (blue). In comparison is the PET reference Raman spectrum obtained from a commercial PET water bottle (black). (**C**) SEM image of the PET found in the deep seawater, which is also the location for SERS measurement.

In seawater samples from Long Beach, CA, we have also found nanostructures with a ball-stick shape surrounded by the co-deposited Ag NP aggregates (Fig. 4A). According to the SERS spectrum, most peaks agree well with those from a benchmark Raman spectrum from polyethylene terephthalate (PET) plastic water bottles (Fig. 4B). Some of these prominent peaks (*45*, *46*) are attributed to the ester C(O)O bending mode at 861 cm^−1^, symmetric C–O–C stretching at 1180 cm^−1^, CH_2_ wagging at 1289 cm^−1^, aromatic ring stretching at 1615 cm^−1^, and C=O stretching at 1726 cm^−1^. It is known that PET can have ball-stick morphology from electrospinning processes (*47*). Some bacteria also have ball-stick shapes, but they would flatten after full dehydration (*48*). To further confirm that the identified NPs were not bacteria, we dehydrated the samples for 2 weeks and observed them under SEM again. The nanoplastic particles remained in the original three-dimensional structures (fig. S8), in contrast to bacteria (*48*). The ball-stick–shaped PET nanoplastics are also found in water from Jeju, South Korea (Fig. 1C).

Unexpectedly, PET nanoplastics are also found in water samples from the offshore location in the Gulf of Mexico, which are collected from 311-m deep under the water surface (blue spectrum in Fig. 4B and SEM in Fig. 4C). It has been reported that plastic particles can quickly sink to the depths of 200 to 800 m due to regional conditions and biofouling, which also influences their density and vertical distribution in the water column (*49*, *50*). In addition, the settling rates of small particles are influenced by factors like salinity (*51*). Furthermore, environmental particles are known to adsorb and/or interact with contaminants, sediments, minerals, organics, and biologicals (such as proteins) on their surfaces, which may also alter their transport (*52*). Wang *et al.* (*53*) investigated the dynamic behavior of a range of polymeric materials with various coatings and observed a gradual descent of PET particles. Therefore, the detection of PET particles in 311-m-deep water is not unreasonable.

It has been reported that polyester is the dominant species among the microplastics found underwater in the sea (*54*), but little is known about the morphological information of the PET nanoplastics in the environments. The PET particles from the deep sea showed irregular shapes, unlike the ball-stick shape from the Long Beach and Jeju locations (Fig. 4A). We note that since the SSBD process was developed for bio-sensing without damaging molecules (*26*), the thermal process in SSBD is unlikely to melt the plastic particles or change their morphologies. In addition, the reference SSBD experiment on laboratory-synthesized PS NP suspension showed the intact shapes of the spherical particles (Fig. 1C). Furthermore, PET has a higher melting of 260°C than that of PS (240°C). Therefore, the different morphologies of the PET particles found in different locations may be due to their different synthesis techniques or how they were fragmented from larger pieces. For the underwater samples, we performed five independent SSBD experiments to test the reproducibility of our results. All deposited spots show PET peaks in the Raman spectrum (fig. S9).

## DISCUSSION

Nanoplastics research is an emerging field (*55*). Toxicological investigations into nanoplastics should address the route of exposure (e.g., inhalation, ingestion, or dermal penetration), concentrations, and particle morphology. Little evidence regarding exposure levels is currently available (*56*), and hurdles with the separation, identification, and quantification of environmental NPs have made appropriate studies difficult to design. While not yet qualitative, our SSBD technique, which provides direct morphological information of nanoplastics, can be a valuable tool that can complement existing quantitative techniques, such as Pyr-GC/MS, to address these challenges. To make the SSBD quantitative, more fundamental fluid mechanics studies of the SSBD deposition process are warranted to establish the relationship between the density of the deposited particles and the concentration of the suspended particles in the solution.

Our goal of the study is to develop a rapid, convenient environmental nanoplastics detection technique in parallel to the existing ones, such as GC/MS, Fourier transform infrared, and Raman spectroscopy. Along with the previous studies, the results suggested a wide distribution and high diversity of nanoplastic particles in the ocean (*42**,* *57*), including the deep ocean, where there is less effect from anthropogenic activities (*49**,* *50*). According to earlier studies, the micro- and nanoplastic particles reach the deep ocean, such as the level of thermocline (approximately 200 to 1000 m from the surface), following the water formation path (*49*) and biofouling processes (*50*). The highly diversified plastic particles identified from various places globally are also in line with the other studies (*52*).

Although the morphological information of nanoplastic particles is beneficial to environmental and toxicological studies, direct observation with nonconcentrated water samples is extremely difficult. The value of the SSBD technique is to efficiently concentrate the substances from the water, which allows us to detect the particles with a relatively small volume of water. This also considerably enhances the sensitivity of the downstream analyses, including SEM, Raman, and EDX. For example, for the detection of PS NPs (density from 0.96 to 1.05 g/cm^3^), the lower detection limit for pyrolysis GC/MS (pyGC/MS) is approximately 100 ng (*58**,* *59*). For the PS particles with 1-μm diameter, the molecular mass is roughly 0.3 × 10^−3^ ng per particle. If there are 10,000 such particles per liter of water, then over 30 liters of water needs to be filtered to obtain 100 ng of particles for the analysis of GC/MS. If the size of the particles is down to 200-nm diameter, then the minimum volume of water that needs to be filtered for GC/MS analysis is 4000 liters with a similar calculation. In contrast, our platform can easily detect particles at a density of ~2 × 10^7^ particles/ml (i.e., 0.1 parts per million) of solution with less than 1 ml of water as demonstrated in this study.

In summary, our study shows that nanoplastics exist widely in the ocean and can be identified with advanced technology. Combined with our unique SSBD method, state-of-the-art analytical tools, including SEM, EDX, and Raman, can be used for the detection and characterization of nanoplastic particles in the environment. The observed nanoplastics comprise various polymers, including nylon, PS, and PET. Their morphologies are highly diverse, including nanofibers, flakes, ball-stick shapes, and other irregular shapes. These are distinctly different from the spherical plastic NPs used in laboratory toxicity studies. Our results should prompt researchers to redesign nanoplastics toxicity studies based on their real morphology and chemistry and explore their origins and evolution processes.

## MATERIALS AND METHODS

### Laboratory preparation

Contamination of samples by laboratory contaminants and airborne particles is a major consideration. To avoid contamination, rigorous methods were adopted. Laboratory staff avoided the use of synthetic clothing, and only cotton laboratory coats were worn. All surfaces were washed with ethanol and Milli-Q water before handling water samples.

### Water sample collection

Clean, unused glass containers were used for water sample collection and storage. Before collection, these containers were washed thoroughly in the laboratory with Milli-Q water. The samples were sealed, and undigested samples were not handled further to avoid contamination. Glass bottles were used directly to collect all seashore samples. The deep ocean samples from the Gulf of Mexico were collected using Niskin sampling bottles (PVC, the spigot is made by PE) attached to a large metal frame and immediately transferred to glass bottles on board the research vessel. The deep water collection is achieved using a conductivity temperature depth (CTD) rosette. Only the water at the designated depth will be collected, and no water from other depths can enter the bottle. Once the CTD rosette was pulled out of the ocean, the water samples were collected in glass bottles, which were precleaned to remove any possible plastic contaminations and tightly covered with sterile aluminum foils before sealing. The unique design ensured that there was no contamination from the shallow water. Although PVC and PE could be potential contaminants in the deep-sea samples, none were found in our SSBD experiments.

### SSBD process

The seawater samples for SSBD were prepared by mixing 800 μl of Ag NP suspension (10 nm, 0.02 mg/ml supplied in 2 mM sodium citrate, Abs. 390 to 400 nm, Thermo Scientific Chemicals) and 200 μl of as-collected seawater. The solution was contained in a quartz cuvette with a glass slide inserted therein (fig. S2). An 800-nm femtosecond pulsed laser (Spectra-Physics, Tsunami) with a repetition rate of 80.7 MHz and a pulse duration of 200 fs was directed to the sample using a 20× objective lens with a numerical aperture of 0.42. The laser is focused on the surface of the glass slide. The laser was illuminated for ~10 s to generate photothermal surface bubbles on the glass substrate. We note that the plasmonic effect produced by Ag NPs with a size of 10 nm, which has a surface plasmon resonance of ~400 nm, is too weak to create a photothermal bubble unless long laser irradiation is used when they were dispersed in deionized water (fig. S10) (*60**,* *61*). However, when mixed with seawater (pH 8.1), Ag NPs agglomerated and the overall light absorbance increased (fig. S11), allowing bubble formation within 10 s. After the surface bubble was generated, it was allowed to grow to ~40 μm in diameter before the laser was cut off, and then the bubble started to shrink. After the bubble shrank and eventually vanished, suspended particles were deposited on the glass substrate, which was taken out of the solution and dried for 2 hours. Raman mapping was conducted on the deposited sample. Because of the co-deposition of colloidal nanoplastics and Ag NPs, the SERS effect is activated. We have also observed microplastic particles in some deposits, but our visualizations and characterizations focused on nanoplastics in this work. We have also confirmed that the as-purchased Ag NPs do not contain any plastics (fig. S4A).

### Electron microscope operating condition

For the SEM imaging, a 3.0-nm Au/Pd layer was coated on the SSBD spots using a sputtering device (ACE600 Carbon & Sputter Coater). Field emission SEM (Magellan 400) was used to acquire the images at a 5-keV accelerating voltage. EDX mappings were conducted at 10 keV (acquisition time: 60 s) using a Bruker EDX system (Bruker Nano GmbH Berlin) on a FIB-SEM (Helios G4 UX, Thermo Fisher Scientific) platform.

### Chemical identification with Raman measurements

Chemical identification of nanoplastics can be characterized by Raman vibrational bands. The spatial resolution (ρ) in this setup (NRS-5100, Jasco, confocal Raman microscope) is diffraction-limited to 532 nm (ρ = 0.61 λ/NA, where λ (= 785 nm) is the wavelength of light and NA (= 0.9) is the numerical aperture of the objective lens). SERS uses an optical electric field in the nanoscale spatial region, which is produced by localized surface plasmon resonance of the metal NPs so that it has the advantage of enhancing the Raman signals of the label-free analytes (*62*). Researchers have already demonstrated that the SERS ability to leverage Ag NPs was effective in detecting engineered nanoplastics (*36*, *37*). Thus, the fingerprint of the nanoplastics was investigated using an Ag-based SERS spot that SSBD produced. SERS mapping was acquired at 1.5-μm intervals using the 785-nm excitation laser with 600 groove/mm grating unless otherwise noted. The excitation power was about 35 mW. The center wave number was fixed at 1300 cm^−1^. The scattered light was detected with a backscattering configuration. Automatic fluorescence correction was applied to all Raman mapping measurements. The Raman system included microfocus with a 100× objective lens. The location of nanoplastics is recorded via an overlay of Raman mapping and an optical image of the SSBD spot taken by a 100× objective lens as shown in fig. S4B. After acquiring the image of the entire SSBD spot through SEM, partitioning it at 1.5-μm intervals as in Raman mapping, we can determine the nanoplastics locations.
