# Supplementary material for: Direct observation and identification of nanoplastics in ocean water
Source: Sci Adv. 2024 Jan 26;10(4):eadh1675. doi: 10.1126/sciadv.adh1675 (PMC10816700; doi:10.1126/sciadv.adh1675)
Supplement: Supplementary file 1 — Table S1 Figs. S1 to S11 [file sciadv.adh1675_sm.pdf]

Science Advances
AAAS

Supplementary Materials for

Direct observation and identification of nanoplastics in ocean water

Seunghyun Moon *et al.*

Corresponding author: Wei Xu, wei.xu@tamucc.edu; Tengfei Luo, tluo@nd.edu

Sci. Adv. **10**, eadh1675 (2024)
DOI: 10.1126/sciadv.adh1675

This PDF file includes:

Table S1
Figs. S1 to S11

Supplementary Table 1. Water collection sites. Seawater samples collected from seven different locations across two oceans. *Surface denotes less than 1.5 m.

<table>
<tr><th colspan="3" rowspan="2">Geographical location</th><th rowspan="2">Latitude/ Longitude</th><th rowspan="2">Water collection depth</th><th colspan="2">Particle detected</th></tr>
<tr><th>SERS detected?</th><th>SEM detected?</th></tr>
<tr><td rowspan="6">USA</td><td rowspan="3">California</td><td rowspan="2">Long Beach: Pier Point Harbor</td><td rowspan="2">33°45'40.0"N/ 118°11'45.6"W</td><td rowspan="2">Surface</td><td>Nylon</td><td>Yes</td></tr>
<tr><td>PET</td><td>Yes</td></tr>
<tr><td>Marina Del Rey: Main channel</td><td>33°57'43.9"N/ 118°27'25.2"W</td><td>Surface</td><td>PS</td><td>No</td></tr>
<tr><td>Massachusetts</td><td>Dartmouth: Apponagansett Bay</td><td>41°35'10.5"N/ 70°57'14.5"W</td><td>Surface</td><td>PS</td><td>No</td></tr>
<tr><td>Texas</td><td>Cole Park</td><td>27°46'24.2"N/ 97°23'19.0"W</td><td>Surface</td><td>PS</td><td>Yes</td></tr>
<tr><td rowspan="2">Gulf of Mexico</td><td rowspan="2">FGRR45 (XR2_2204)</td><td rowspan="2">27°45'57.6"N/ 93°37'48.0"W</td><td>Surface</td><td>PS</td><td>No</td></tr>
<tr><td></td><td>311 m</td><td>PET</td><td>Yes</td></tr>
<tr><td rowspan="2">South Korea</td><td>Ulsan</td><td>Daewangam Park</td><td>35°29'21.1"N/ 129°26'23.5"E</td><td>Surface</td><td>PS</td><td>No</td></tr>
<tr><td>Jeju Island</td><td>Yongduam Rock</td><td>33°30'58.9"N/ 126°30'43.6"E</td><td>Surface</td><td>PET</td><td>Yes</td></tr>
<tr><td rowspan="2">China</td><td rowspan="2">Shenzhen</td><td rowspan="2">Longgang</td><td rowspan="2">22°36'12.3"N/ 114°21'26.3"E</td><td rowspan="2">Surface</td><td>PS</td><td>No</td></tr>
<tr><td>Unidentified</td><td>Nanofiber</td></tr>
</table>

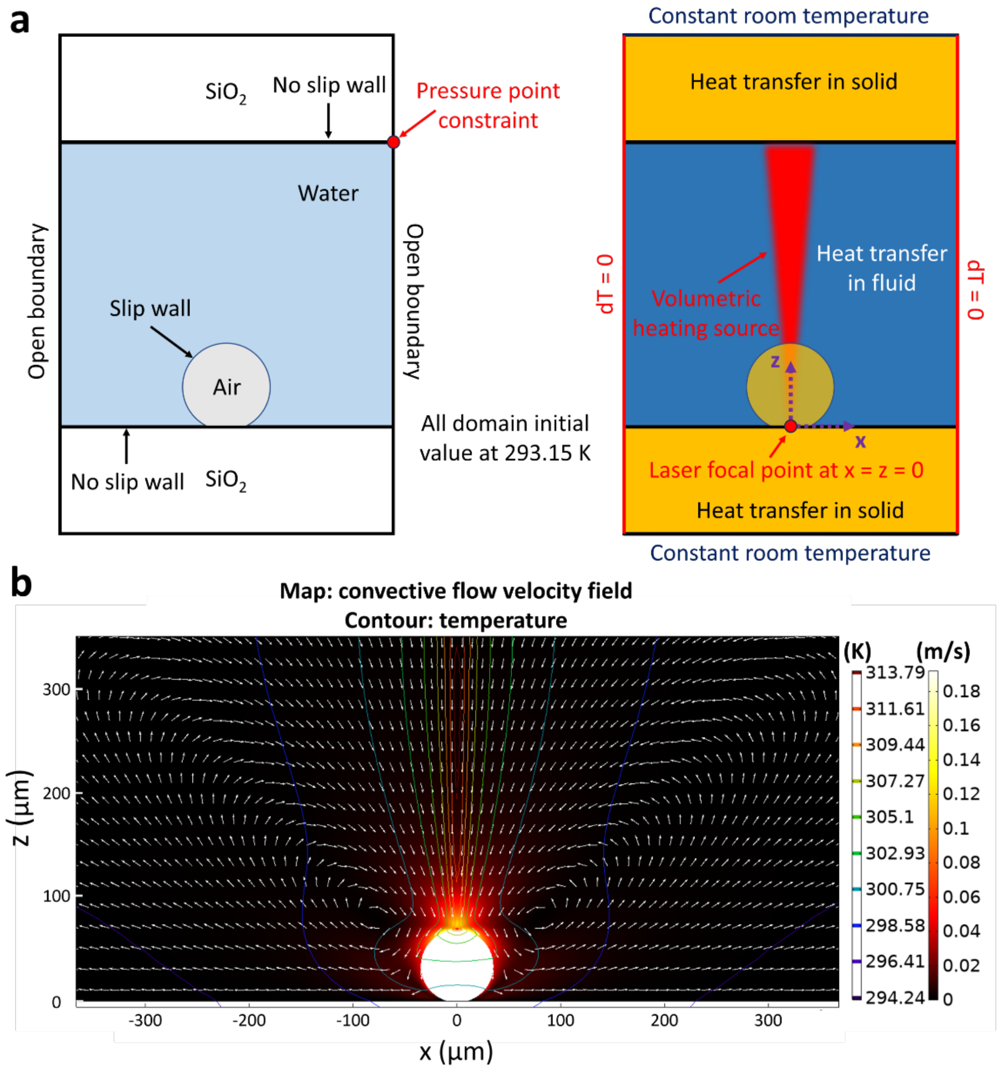

Supplementary Fig. 1. Computational fluid dynamics simulation results of the steady-state flow field around a bubble on a glass substrate in the plasmonic suspension subject to laser beam heating. (a) Left: the schematic model and boundary conditions for the finite-element thermofluidic simulation. Right: the heating profile due to laser irradiation. (b) The simulation illustrates the thermo-capillary flow pattern around the plasmonic surface bubble induced by volumetric heating from the laser in the suspension. Arrows indicate the flow direction, while solid lines represent the isothermal temperature contours. The bubble has a diameter of ~30 um, and the laser has a power of 500 mW. This is a 2D simulation, but the result is generalizable to 3D geometry. Mass, momentum and energy equations are solved in the COMSEL Multiphysics package using the finite element method. The flow is in the laminar flow regime, and the bubble is treated as a non-fluidic rigid body, but the surface of the bubble (gas/water boundary) has a slip boundary condition with the Marangoni effect considered. The interface between the liquid and the glass substrate uses the non-slip boundary condition. The domain of the system is divided into 2×10^6 finite elements. A more detailed study of the thermofluidic flow around the surface bubble can be found in Ref. (*28*).

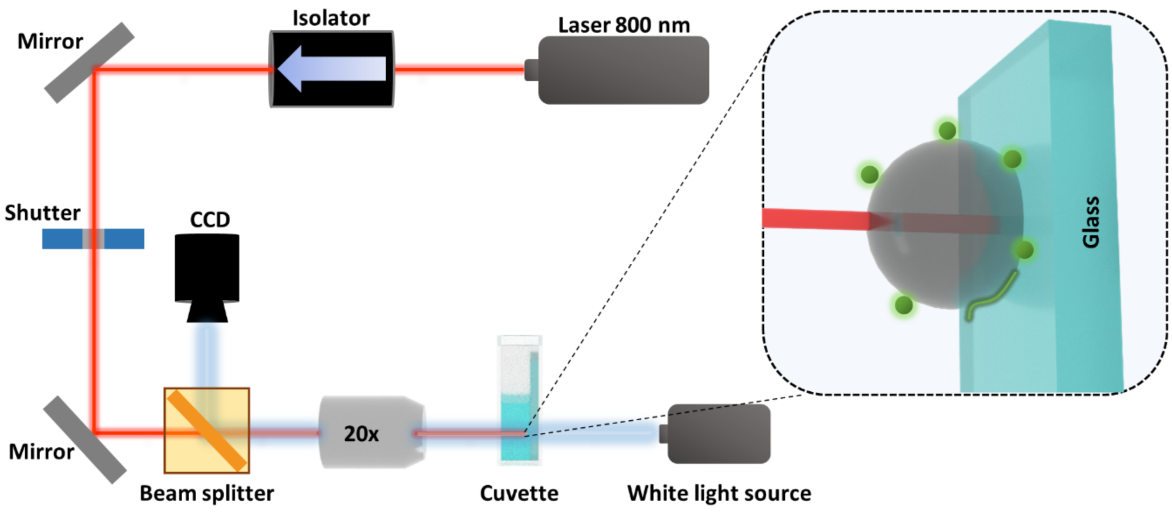

Supplementary Fig. 2. Optical setup for SSBD process. Seawater samples for SSBD were prepared by mixing Ag NP suspension with collected seawater. This mixture was placed in a quartz cuvette, and an 800 nm femtosecond pulsed laser was focused on the glass slide through a 20× objective lens. The growth of bubble was monitored using a CCD camera, and the laser was stopped using a shutter when the bubble reached a size of about 40 μm.

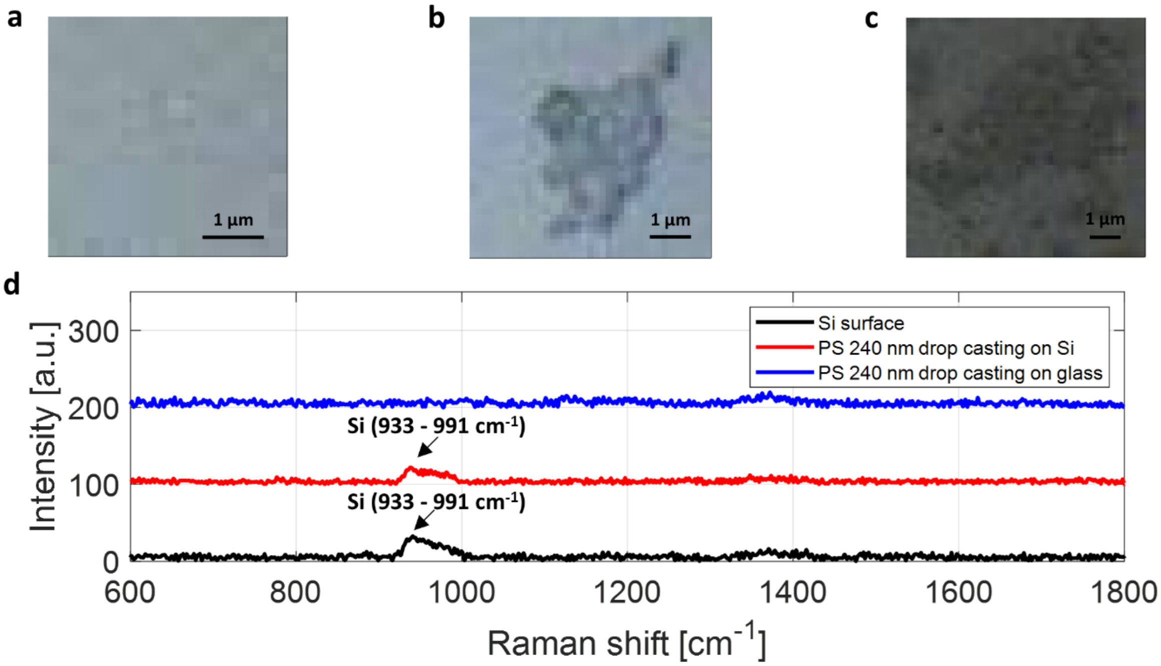

Supplementary Fig. 3. Raman analysis of PS on Si and glass substrates without SERS enhancement. (a) Optical microscope image of a Si substrate. (b) Drop casting of an engineered PS (240 nm) solution with a relatively high concentration of 5 ppm on the Si substrate. (c) Drop casting of the PS (240 nm) solution (5 ppm) on a glass substrate. (d) Raman spectra of bare Si surface, PS on Si and PS on glass. No PS Raman peaks could be observed from these spectra due to the lack of the SERS effect. Only silicon peaks could be observed. With SERS enabled by SSBD, we could detect PS with a concentration of 0.1 ppm (see Supplementary Fig. 4).

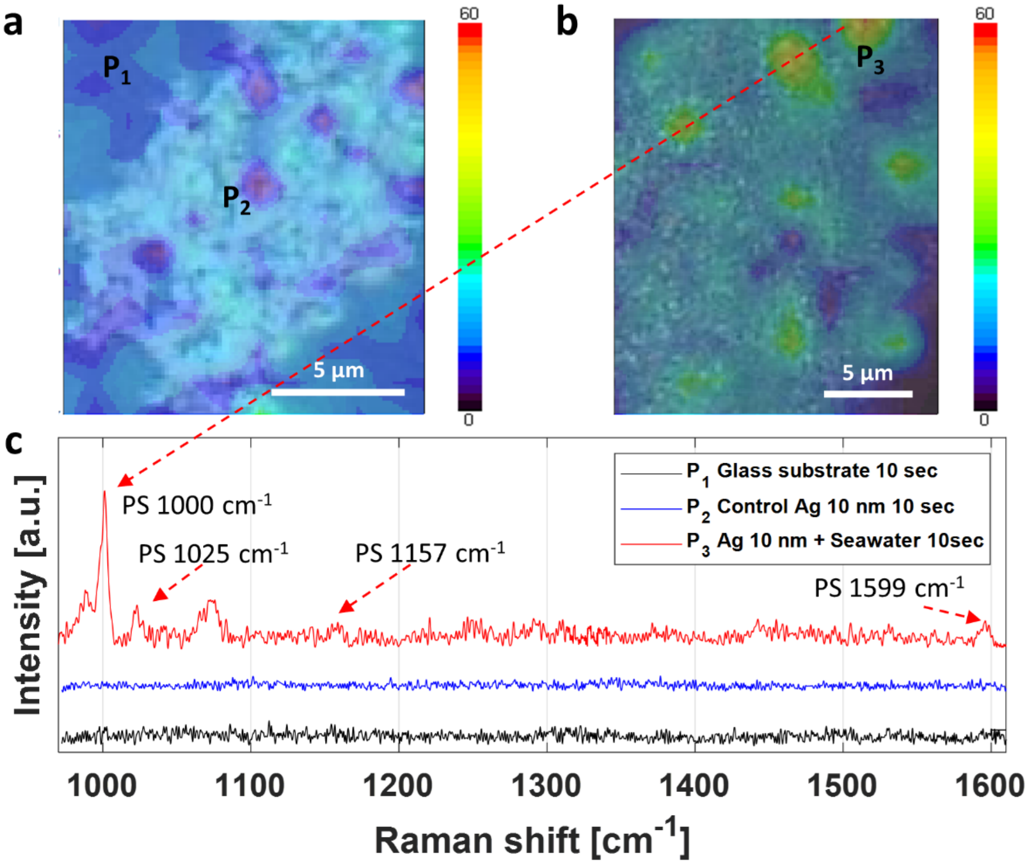

Supplementary Fig. 4. Validation of Ag NPs Solution. (a) Overlay of optical image of control sample and Raman mapping at 1000 cm^{-1} with 1200-groove/mm grating. The control sample was fabricated by the SSBD process using pure Ag NP 10 nm suspension without seawater. NaCl solution (3 wt%) was added into the Ag suspension to aggregate Ag NPs for bubble generation. (b) SSBD spot deposited using the mixture suspension of Ag NP and seawater. It shows the overlay image of the SSBD spot and Raman mapping at 1000 cm^{-1}. (c) Typical SERS spectra detected from the seawater and control samples. From the control Ag NP sample and glass substrate, only weak fluorescence signals were observed between 1300 and 1400 cm^{-1}, while that from seawater shows obvious PS peaks.

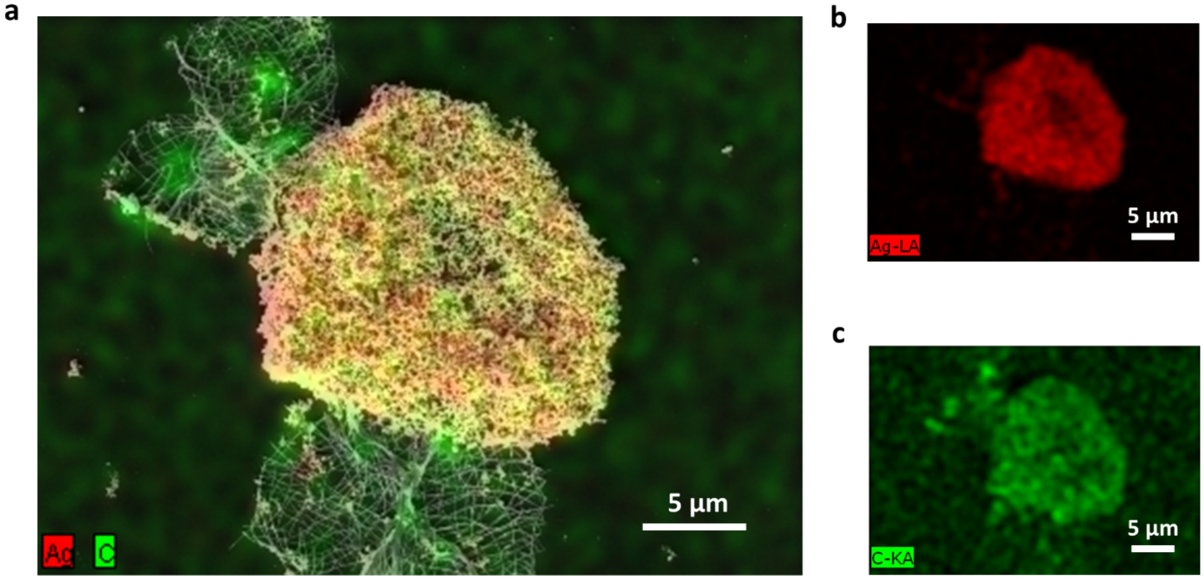

Supplementary Fig. 5. Observation of carbon-based nanofibers. (a) For the sample from Shenzhen, China, EDX elemental mapping overlaid with the SEM image shows the nanofibers are of a carbon nature. Red and green colors represent Ag and C, respectively. (b) Individual color map of Ag. (c) Individual color map of C. While the nanofibers are likely of plastic nature given their morphology and carbon nature, our Raman spectrum could not pick up obvious peaks belonging to Nylon.

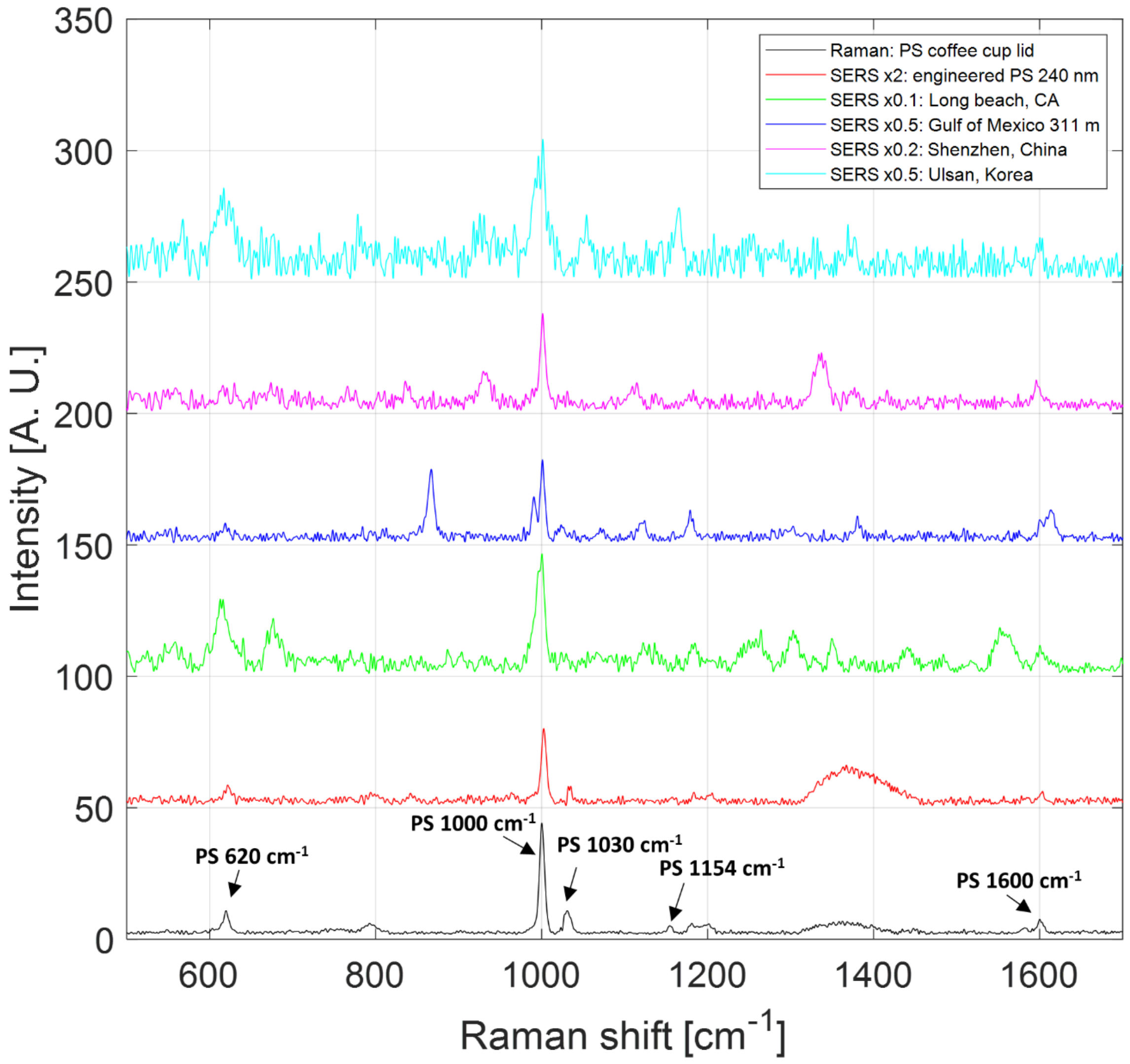

Supplementary Fig. 6. Raman spectra of a reference PS sample and water samples from different locations. Black line: PS Raman spectrum obtained from a coffee-cup lid. Raman exposure time was 10 s, and data acquisition was performed 10 times at one location to increase the signal-to-noise ratio. Red line: SERS spectrum of engineered PS NPs (240 nm, 0.1 ppm) co-deposited with Ag NPs (10 nm) by SSBD. Green (Long Beach, CA), blue (Gulf of Mexico), magenta (Shenzhen, China), and cyan (Ulsan, Korea) lines are SERS spectra of seawater obtained from different sites, but no PS particles could be observed from SEM in these samples.

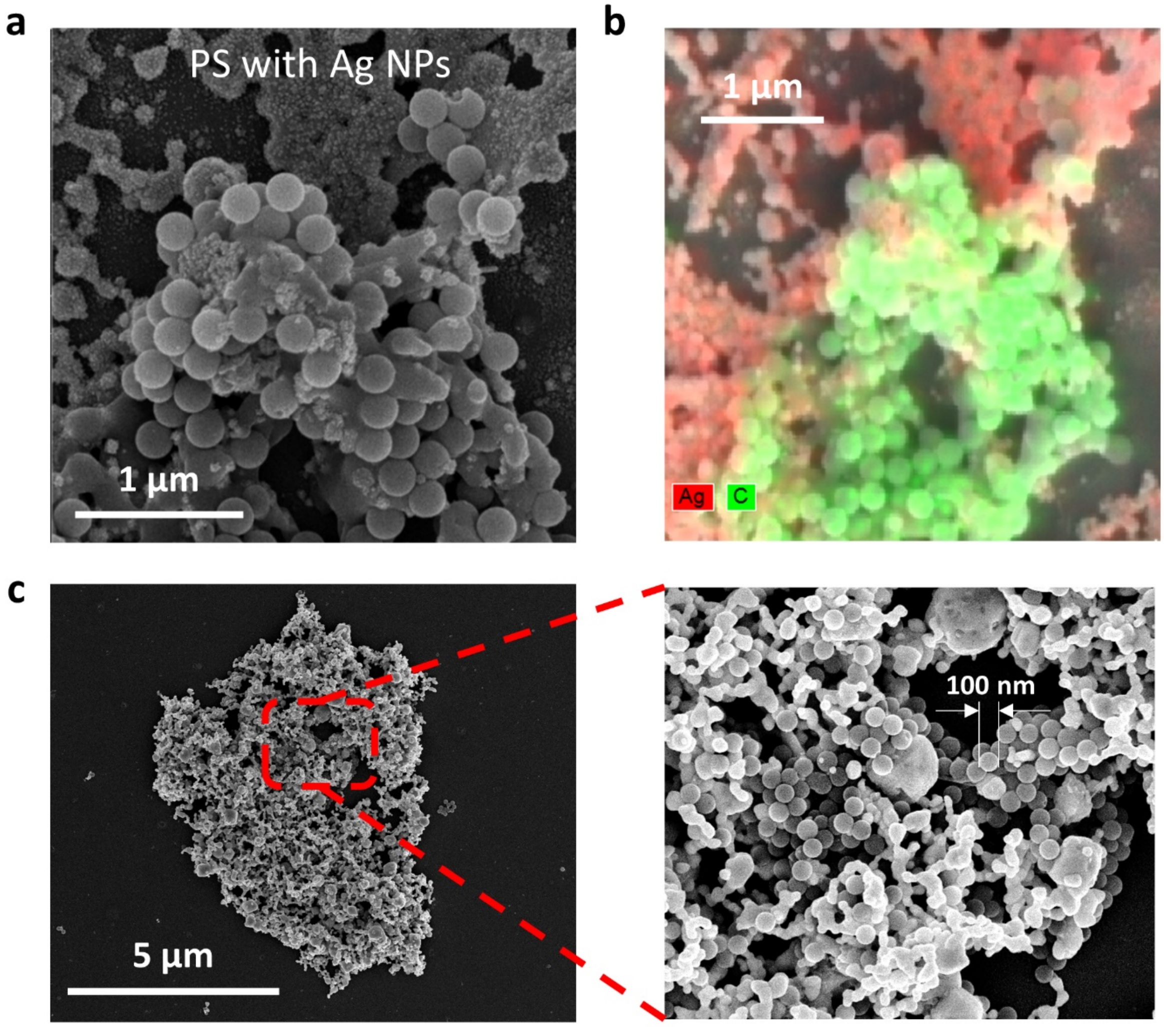

Supplementary Fig. 7. Validation tests of SSBD using lab-synthesized PS nanoplastics in deionized water and seawater. (a) Co-deposition of engineered PS NPs and Ag NPs by SSBD. All particles were dispersed in deionized water and 0.1 PPM of engineered PS NPs solution was used. (b) EDX mapping of the SSBD spot. Green and red colors represent C and Ag signals, respectively. (c) Co-deposition of engineered PS NPs (100 nm in diameter) and Ag NPs dispersed in seawater (400 µL). The concentration of the PS NPs was 1 x 10^9 particles/mL. (Right image) Enlarged SEM image.

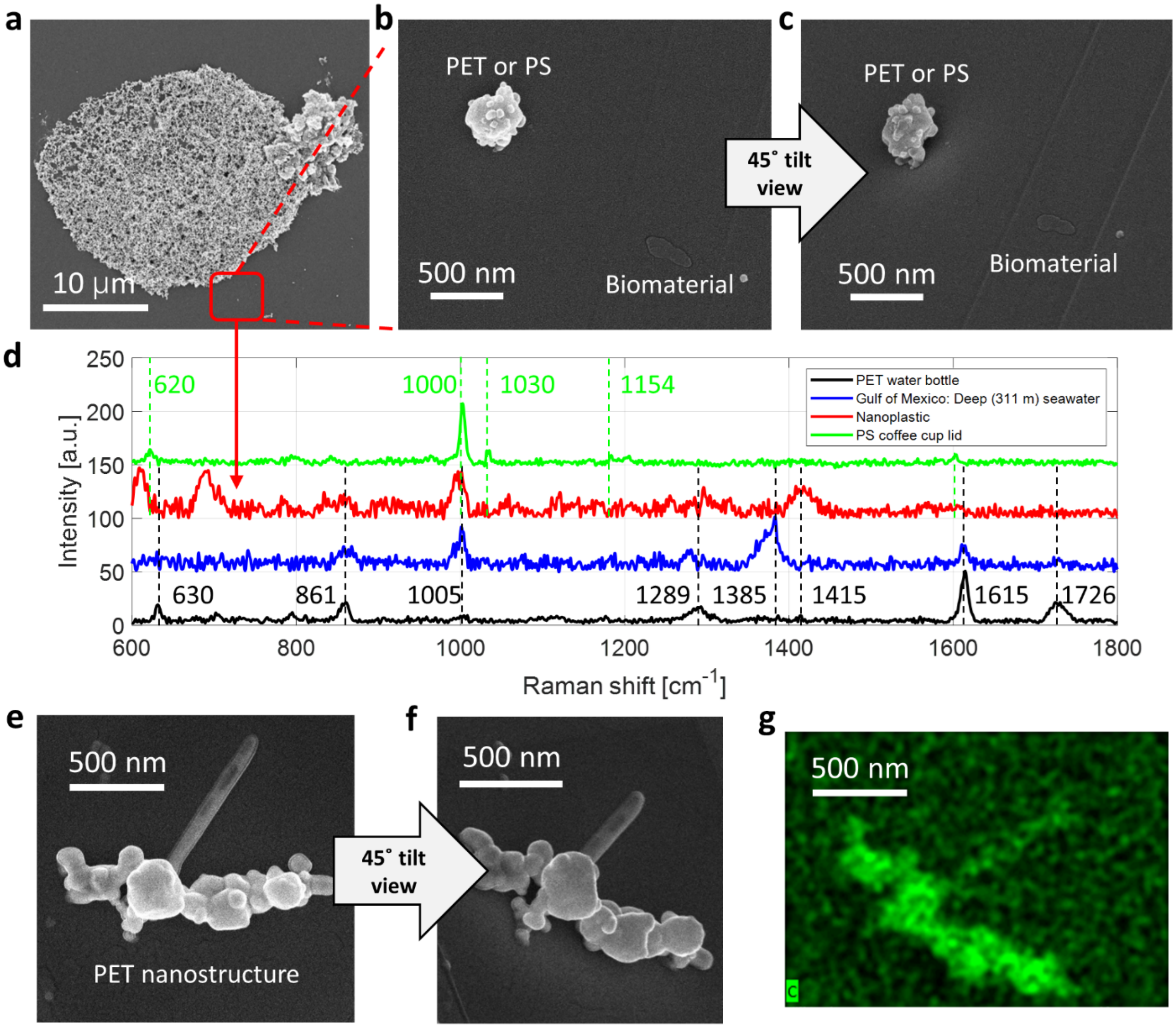

Supplementary Fig. 8. **Dehydration test.** (a) SEM image of SSBD deposition spot from the 311 m-deep seawater sample from the Gulf of Mexico. (b) Exterior morphologies of a nanoplastic particle and a biomolecule. SEM images were taken after fully drying the sample for 2 weeks in the ambient condition. (c) The SEM sample stage was tilted at 45° to observe the morphological difference between the two materials. (d) Comparison of Raman spectra. The red line indicates the SERS spectrum obtained from particles shown in (b). Although the spectrum resembles the PET sample obtained from the Gulf of Mexico (blue line), it also contains some of the characteristics of PS (green line), so it was classified as unidentified nanoparticles and can be a PET particle with PS oligomers attached to it. (e) Top view of another PET nanostructure from the Long Beach sample. (f) Tilted view of the sample (e). (g)EDS carbon mapping of (e).

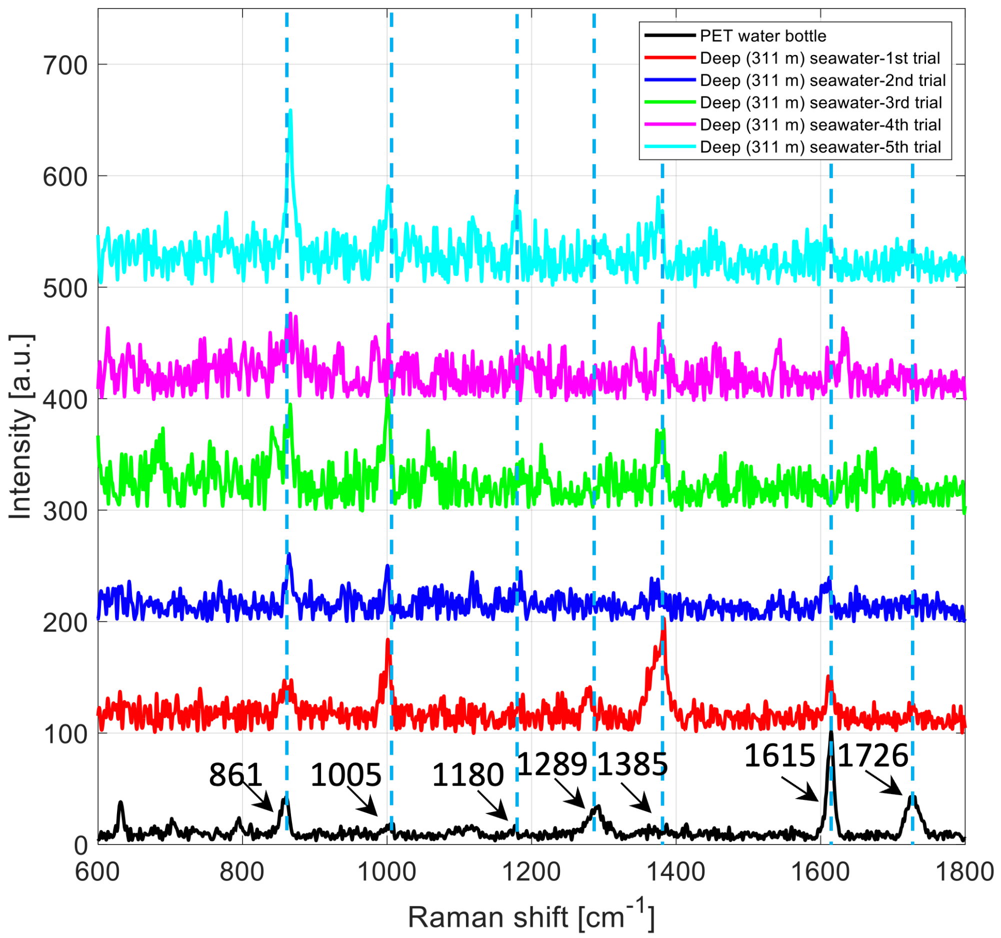

Supplementary Fig. 9. SERS spectra of five independent SSBD experiments on the 311 m-deep seawater sample from the Gulf of Mexico. Each experiment uses 200 μL of seawater sample. PET spectra were obtained in all experiments.

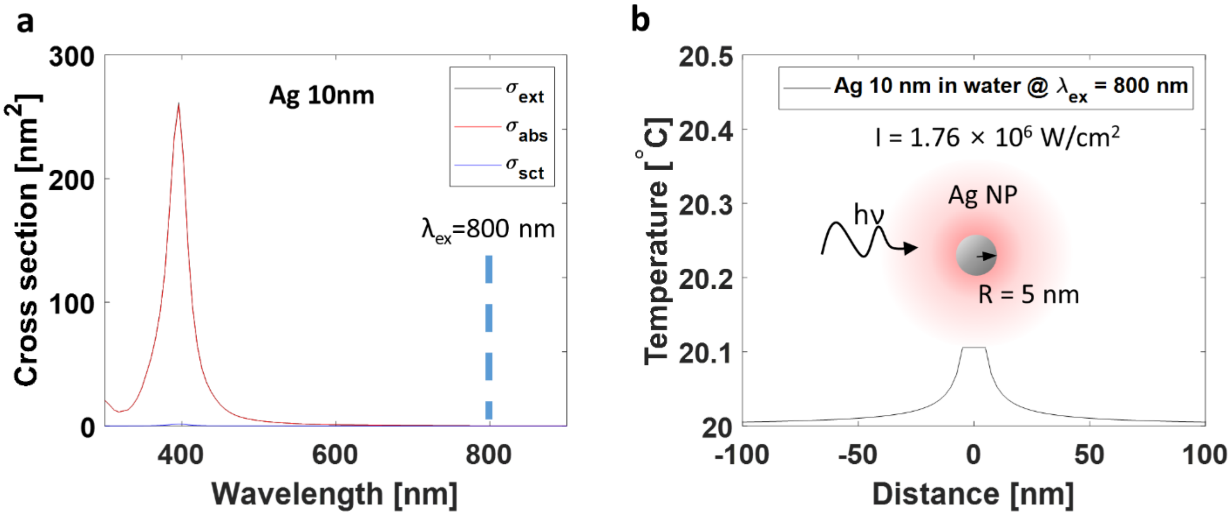

Supplementary Fig. 10. Optical cross-sections and temperature profile of Ag NP. (a) The calculated optical cross-sections of an Ag NP. **b**, Temperature profile of a single Ag NP in water environment. The optical cross-sections (σ) of the Ag NP were calculated by COMSOL Multiphysics. (b) A spherical NP with a radius (*R*) is surrounded by water domain including the Perfect Matching Layer (PML). The PML layer absorbs all outgoing wave energy without impedance mismatch that can cause stray reflections at the boundary. To estimate the temperature profile of the Ag NP in the water (Fig. S1b) at the given experimental excitation wavelength, the analytical solution for the 1-D heat conduction equation is used*(51, 52)*:

$$T(r) = T\left(\infty\right) + \frac{P_0}{4\pi\kappa_{water}r} \text{ for } r > R, \quad \text{(S1)}$$

where $T(\infty)$ [K] is ambient temperature, P_0 is the heat power dissipated in the NP, κ_{water} is the thermal conductivity of water, *R* is the radius of the NP, and *r* is the radial coordinate. The heating power dissipated in the NP is given by $P_0 = \sigma_{abs} I$, where σ_{abs} is the absorption cross-section of NP at the excitation wavelength obtained from Fig. S1a and *I* (= 1.76 × 10^6 W/cm^2) is the power density of the excitation laser.

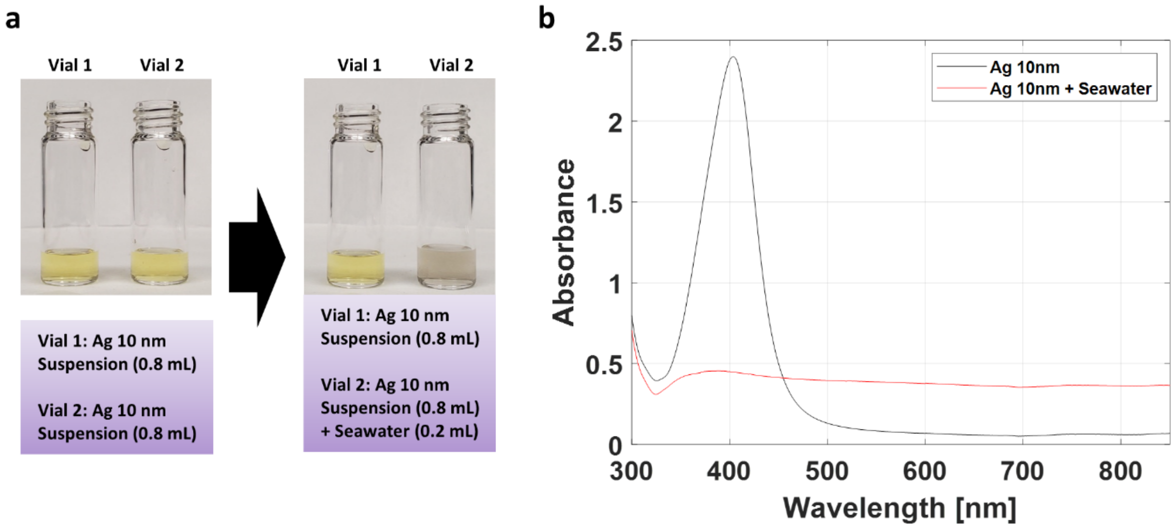

Supplementary Fig. 11. Effect of seawater on Ag NP suspension. (a) Ag NP (10 nm) suspension before and after mixing with seawater. Mixing with seawater changes the suspension to a darker color. (b) Measured absorbance of the pure Ag NP suspension and the Ag NP suspension mixed with seawater. As seawater water is added into the Ag NP suspension, the resonance peak at about 400 nm disappeared due to the aggregation of Ag NPs and the overall absorbance increased, resulting in sufficient laser-to-heat conversion and thus surface bubble generation.
